# First-in-Human Study of [^68^Ga]Ga-NODAGA-E[c(RGDyK)]_2_ PET for Integrin α_v_β_3_ Imaging in Patients with Breast Cancer and Neuroendocrine Neoplasms: Safety, Dosimetry and Tumor Imaging Ability

**DOI:** 10.3390/diagnostics12040851

**Published:** 2022-03-30

**Authors:** Malene Martini Clausen, Esben Andreas Carlsen, Camilla Christensen, Jacob Madsen, Malene Brandt-Larsen, Thomas Levin Klausen, Søren Holm, Annika Loft, Anne Kiil Berthelsen, Niels Kroman, Ulrich Knigge, Andreas Kjaer

**Affiliations:** 1 Department of Clinical Physiology and Nuclear Medicine & Cluster for Molecular Imaging, Copenhagen University Hospital—Rigshospitalet & Department of Biomedical Sciences, University of Copenhagen, 2100 Copenhagen, Denmark; malene.martini.clausen@regionh.dk (M.M.C.); esben.andreas.carlsen.01@regionh.dk (E.A.C.); cch@minervaimaging.com (C.C.); jacob.madsen@regionh.dk (J.M.); bdqdk@leo-pharma.com (M.B.-L.); thomas.levin.klausen@regionh.dk (T.L.K.); soeren.holm@regionh.dk (S.H.); annika.loft.jakobsen@regionh.dk (A.L.); anne.kiil.berthelsen@regionh.dk (A.K.B.); 2 Department of Oncology, Copenhagen University Hospital—Rigshospitalet, 2100 Copenhagen, Denmark; 3 ENETS Neuroendocrine Tumor Center of Excellence, Copenhagen University Hospital—Rigshospitalet, 2100 Copenhagen, Denmark; ulrich.peter.knigge@regionh.dk; 4 Department of Breast Surgery, Copenhagen University Hospital—Herlev/Gentofte Hospital, 2730 Herlev, Denmark; niels.kroman@regionh.dk; 5 Departments of Clinical Endocrinology and Surgical Gastroenterology, Copenhagen University Hospital—Rigshospitalet, 2100 Copenhagen, Denmark

**Keywords:** first in human, PET, RGD, breast cancer, neuroendocrine neoplasm, alphavbeta3 integrin

## Abstract

Arginine-Glycine-Aspartate (RGD)-recognizing cell surface integrins are involved in tumor growth, invasiveness/metastases, and angiogenesis, and are therefore an attractive treatment target in cancers. The subtype integrin α_v_β_3_ is upregulated on endothelial cells during angiogenesis and on tumor cells. In vivo assessment of integrin α_v_β_3_ is possible with positron emission tomography (PET). Preclinical data on radiochemical properties, tumor uptake and radiation exposure identified [^68^Ga]Ga-NODAGA-E[c(RGDyK)]_2_ as a promising candidate for clinical translation. In this first-in-human phase I study, we evaluate [^68^Ga]Ga-NODAGA-E[c(RGDyK)]_2_ PET in patients with neuroendocrine neoplasms (NEN) and breast cancer (BC). The aim was to investigate safety, biodistribution and dosimetry as well as tracer uptake in tumor lesions. A total of 10 patients (5 breast cancer, 5 neuroendocrine neoplasm) received a single intravenous dose of approximately 200 MBq [^68^Ga]Ga-NODAGA-E[c(RGDyK)]_2_. Biodistribution profile and dosimetry were assessed by whole-body PET/CT performed at 10 min, 1 h and 2 h after injection. Safety assessment with vital parameters, electrocardiograms and blood tests were performed before and after injection. In vivo stability of [^68^Ga]Ga-NODAGA-E[c(RGDyK)]_2_ was determined by analysis of blood and urine. PET images were analyzed for tracer uptake in tumors and background organs. No adverse events or pharmacologic effects were observed in the 10 patients. [^68^Ga]Ga-NODAGA-E[c(RGDyK)]_2_ exhibited good in vivo stability and fast clearance, primarily by renal excretion. The effective dose was 0.022 mSv/MBq, equaling a radiation exposure of 4.4 mSv at an injected activity of 200 MBq. The tracer demonstrated stable tumor retention and good image contrast. In conclusion, this first-in-human phase I trial demonstrated safe use of [^68^Ga]Ga-NODAGA-E[c(RGDyK)]_2_ for integrin α_v_β_3_ imaging in cancer patients, low radiation exposure and favorable uptake in tumors. Further studies are warranted to establish whether [^68^Ga]Ga-NODAGA-E[c(RGDyK)]_2_ may become a tool for early identification of patients eligible for treatments targeting integrin α_v_β_3_ and for risk stratification of patients.

## 1. Introduction

Cell surface adhesion receptors of the integrin superfamily play a fundamental role in physiological as well as pathophysiological processes. The subfamily of Arginine-Glycine-Aspartate (RGD)-recognizing integrins has drawn most attention in the efforts of producing targeting agents due to implications on several of hallmarks of cancer—tumor growth, invasiveness and metastases and angiogenesis [1]. Integrins consist of one alpha and one beta subunit, where the RGD-recognizing integrins are α_v_β_1_, α_v_β_3_, α_v_β_5_, α_v_β_6_, α_v_β_8_, α_5_β_1_, α_8_β_1_ and α_IIb_β_3_. Targeting particularly integrin α_v_β_3_ has been pursued due to significant upregulation on activated endothelial cells during angiogenesis, but absence on quiescent endothelial cells [2], thus linking it to neo-angiogenesis. Integrin α_v_β_3_ expression is also seen on tumor cells in certain cancers, and the overexpression of integrin α_v_β_3_ may therefore have implications to several cancer entities, e.g., breast, glioblastoma, and prostate [2].

Initial clinical trials with the α_v_β_3_/α_v_β_5_-targeting ligand cilengitide showed a modest effect on tumor growth [3,4], while later phase II/phase III trials failed to meet expectations due to unintended pro-angiogenic effects at lower concentrations, and anti-angiogenetic effect was seen only at higher concentrations [5]. Recently, new promising pure α_v_β_3_ ligands (TDI-4161 and TDI-3761) have been shown to circumvent the pro-angiogenetic effect previously seen with cilengitide [6], hence reinforcing the need for development of methods to assess in vivo the level of α_v_β_3_ integrin expression for selection of patients for such targeted therapies. 

A large number of PET tracers with the RGD motif have been developed and tested preclinically. However, only a few have reached testing in clinical trials, and none have yet been employed for routine clinical use. The clinical translation of first-generation integrin-targeting PET tracers with ^18^F, e.g., ^18^F-Galacto-RGD, was hampered by complex radiochemistry. Thus, several approaches to optimize the production and tracer stability as well as tumor-binding properties have been investigated [7]. Addressing the need for assessing integrin α_v_β_3_, our group evaluated a number of PET tracers utilizing cyclic RGD, either as monomer or dimer coupled with ^64^Cu or ^68^Ga [8,9,10,11,12]. Of these, [^68^Ga]Ga-NODAGA-E[c(RGDyK)]_2_ was found to be particularly promising as it fulfilled the requirements of fast and reliable radiochemical production, imaging abilities, i.e., stable tumor retention and favorable tumor-to-background ratio, favorable human radiation estimates, and was correlated with gene expression of integrin [8,9,10,13,14]. Furthermore, in vitro assessment of the affinity of NODAGA-E[c(RGDyK)]_2_ towards integrin subtype α_v_β_3_ showed an excellent affinity (KD) of 0.075 nM. (Bentsen et al., unpublished data). Taken together, [^68^Ga]Ga-NODAGA-E[c(RGDyK)]_2_ therefore was chosen for human translation. 

Here, we present our single-center first-in-human phase I study of the PET tracer [^68^Ga]Ga-NODAGA-E[c(RGDyK)]_2_ in 10 patients with either breast cancer (BC) or neuroendocrine neoplasms (NEN). The primary objective was to evaluate biodistribution, dosimetry and safety and the secondary objective was to assess tumor uptake. 

## 2. Materials and Methods

### 2.1. Patients

A total of 10 patients older than 50 years with histopathologically confirmed BC or NEN were enrolled in the phase I study from 24 November 2016 to 26 June 2017. All patients gave written informed consent before inclusion. This study was approved by the Danish Health and Medicine Authority (EudraCT no. 2015-005335-41) and the Regional Scientific Ethical Committee (H-16034365) and registered at ClinicalTrials.gov (NCT02970786). This study was performed in accordance with Good Clinical Practice (GCP), and independently monitored by the GCP unit of the Capital Region of Denmark. 

**Figure 1:**
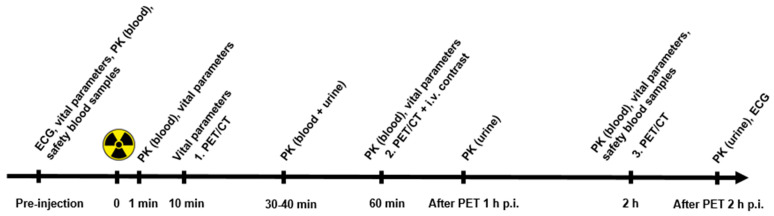
Trial events before and after a single-dose injection of [^68^Ga]Ga-NODAGA-E[c(RGDyK)]_2_. Timeline denotes injection, acquisition of PET/CT imaging, assessment of vital parameters, and collection of blood and urine. Abbreviations: ECG (electrocardiogram), PK (pharmacokinetics), and PET/CT (positron emission tomography).

Patients fasted 4 h before intravenous injection of [^68^Ga]Ga-NODAGA-E[c(RGDyK)]_2_ followed by sequential whole-body PET/CT scans. Two peripheral intravenous catheters were placed, one for tracer injection and one in the contralateral arm for withdrawal of blood samples and administration of i.v. contrast agent. Electrocardiogram and vital signs as well as safety blood samples were monitored before and following [^68^Ga]Ga-NODAGA-E[c(RGDyK)]_2_ injection. In a subset of patients, blood samples were collected after [^68^Ga]Ga-NODAGA-E[c(RGDyK)]_2_ injection for pharmacokinetic analyses including ligand stability. Furthermore, urine was collected for pharmacokinetic analysis and dosimetry in a subset of patients (Figure 1). 

### 2.2. Synthesis of [^68^Ga]Ga-NODAGA-E[c(RGDyK)]_2_

NODAGA-E[c(RGDyK)]_2_ acetate was obtained from ABX GmbH (Radeberg, Germany). All reagents and cassettes were purchased from Eckert and Ziegler (Berlin, Germany). Gallium-68 (T1/2 = 68 min; Emax, β+ = 1.90 MeV (89%)) labelling of NODAGA-E[c(RGDyK)]_2_ acetate was performed in a Modular-Lab Pharmtracer module (Eckert and Ziegler) using a 68Ge/68Ga generator (Galliapharm, 50 mCi, Eckert and Ziegler). The generator was eluted with 6 mL 0.1M HCl. The eluate was concentrated on a Bond Elut SCX cartridge and eluted with 700 µL 5M NaCl/5.5M HCl (41:1). NODAGA-E[c(RGDyK)]_2_ (50 µg, 30 nmol) was labelled in 1000 µL 1.4 M NaOAc buffer pH 4.5 and 400 µL 50% EtOH at 60 °C for 300 s. The resulting mixture was transferred to a Sep-pak C2 light cartridge and washed with saline. [^68^Ga]Ga-NODAGA-E[c(RGDyK)]_2_ was eluted with 1 mL 50% EtOH through a sterile filter and formulated with saline. The synthesis time was 20 min and 533 ± 167 MBq [^68^Ga]Ga-NODAGA-E[c(RGDyK)]_2_ was obtained. See Supplementary Material (Section S1) for a description of the quality control. 

### 2.3. Plasma Pharmacokinetics and Urine Metabolite Analysis

The activity concentration of the urine and plasma was counted on Cobra II TM Gamma Counter (Packard, CT, USA). Blood and urine samples were analyzed on a Dionex UltiMate 3000 column-switching high-performance liquid chromatography (HPLC) system with a Posi-RAM Module 4 (ThermoFisher Scientific, Waltham, MA, USA). The full blood samples were centrifuged (3500 rpm, 4 min) and the supernatant plasma was collected and filtered through a 0.45 μM syringe filter prior to the HPLC analysis [15]. The HPLC analysis consisted of an extraction step and an analytical step, as previously described [16]. During the extraction step, the plasma samples were passed through a shim-pack XR-ODS (30 × 4.6 mm, 2.2 μm). The valves were switched, and the sample was then analyzed on an Onyx monolithic column (C18, 50 × 4.6 mm). The mobile phase for the extraction step was 0.1% trifluoroacetic acid (TFA) in H_2_O, while the analytical step was a gradient method with solvent A (0.1% TFA in MeCN:H_2_O 10:90) and solvent B (0.1% TFA in MeCN:H_2_O 90:10), both with a flow of 1 mL/min. Gradient: 0–6 min (extraction), 6–7 min 5% B, 7–12 min 5–35% B, and 12–14 min 35–5% B. 

### 2.4. PET/CT Acquisition and Image Analysis

Data acquisition was performed using a Biograph mCT PET/CT system (Siemens Medical Solutions, Erlangen, Germany) with an axial field of view of 216 mm. Whole-body PET/CT scans were acquired at 10 min, 1 h and 2 h after intravenous injection of approx. 200 MBq [^68^Ga]Ga-NODAGA-E[c(RGDyK)]_2_. PET/CT scans were obtained in 3D mode with acquisition time of 2 min per bed position (1 min/bed position for lower extremities). A diagnostic CT was obtained before PET 1 h p.i. with a 2 mm slice thickness, 120 kV, and a quality reference of 225 mAs modulated by the Care Dose 4D automatic exposure control system (Siemens Medical Solutions). An automatic injection system was used to administer 75 mL of an iodine-containing contrast agent (Optiray 300; Covidien, Dublin, Ireland) with a scan delay of 60 s and flow rate of 1.5 mL/s, followed by an injection of 150 mL NaCl with a flow rate of 2.5 mL/s. A low-dose CT scan, 2 mm slice thickness, 120 kV, and 40 mAs, was acquired before PET 10 min and PET 2 h p.i., and used for attenuation correction. Using the corresponding CT-scan for attenuation- and scatter correction, the PET data were reconstructed iteratively using the TrueX algorithm including point-spread function and time-of-flight information (Siemens Medical Solutions); the settings were 2 iterations, 21 subsets, 2 mm Gaussian filter, and a 400 × 400 matrix. Pixel size in the final reconstructed PET image was approx. 2 × 2 mm with a slice thickness of 2 mm.

### 2.5. Tumor Uptake by Visual Image Analysis and Activity Quantification

PET/CT analysis was performed by a team of two experienced board certified specialists in nuclear medicine and radiology, respectively. A volume of interest (VOI) was drawn to encompass the entire lesion on PET images, and standardized uptake values (SUV) for primary and metastatic lesions were registered. If a lesion was not visible on PET, the co-registered CT was used for delineation of the tumor. The lesion in each organ with the highest SUV_max_ was reported. Tumor uptake was also qualitatively described as homogeneous or heterogeneous. Tumor size was measured by largest diameter on CT. 

### 2.6. Dosimetry

Dosimetry was based on the decay-uncorrected image sets from the 3 time points (10 patients) supplemented with sampled urine data (7 patients). For each patient, organ, and time point, tissue activity concentration (kBq/mL) was determined in VOIs defined on CT. Activity (per patient, organ and time) was estimated by multiplying concentration values by organ masses of the OLINDA male adult phantom [17], normalized per injected MBq and scaled for patient weight. Time integrated activity coefficients (TIAC, unit h) for each patient and organ was determined by numerical integration and analytical extrapolation to infinity assuming only physical decay. The resulting organ TIACs were averaged over patients. All data were entered into OLINDA/EXM 2.0 software (Vanderbilt University, TN, USA and HERMES Medical Solutions, Stockholm, Sweden) [18]. 

Urine was collected immediately after each scan in pre-weighted plastic bottles. The cumulated decay-corrected activity (MBq) of the excreted urine was plotted over time for all 7 subjects and data fitted to a one-phase exponential association. The resulting limit and half-life were used as input to the bladder voiding model of OLINDA using a bladder voiding interval of 1 h. A detailed description of the dosimetry is available in Supplementary Material (Section S2). 

### 2.7. Histology

Specimens from primary tumor or metastases were obtained from patients undergoing surgery within four weeks of the PET/CT. The specimens were placed in formalin and paraffin embedded within 24 h. The samples were cut in sections of 4 µM and dewaxed through xylene to tap water. For antigen retrieval the sections were treated with proteinase K for 5 min. This was followed by a blocking step with Peroxidase-Blocking Solution (Agilent, S2023) and pre-incubation in 2% BSA for 10 min. For visualizing the intensity and distribution of integrin, α_v_β_3_ sections were incubated with primary antibody (Absolute Antibodies, Ab00890-23.0) in a 1:50 dilution in 2% BSA overnight at 40 °C [19].

For visualization, the sections were incubated with Envision+ system Anti-Rabbit (Agilent, K4003) for 45 min followed by incubation with DAB+ system (Agilent, K3468) for 10 min. Counterstaining was performed with Mayer’s Hematoxylin. The sections were visually evaluated regarding α_v_β_3_ intensity. 

### 2.8. Statistics

Data are presented as the mean with the standard error of mean (SEM) unless otherwise stated. The significance of differences in vital signs and blood tests was evaluated using ANOVA. A *p*-value < 0.05 was considered statistically significant. 

## 3. Results

### 3.1. Patient Characteristics

Ten patients were included in this study—five patients with NEN and five patients with BC. Patient characteristics are shown in Table 1. All patients were, independently of this study, planned for surgical removal of tumor or metastases subsequent to PET/CT; however, one patient turned out to be unresectable, and one patient had known metastatic disease, and was diagnosed with metastatic spinal cord compression shortly after PET/CT and therefore was not a candidate for surgery.

### 3.2. Radiochemistry

All preparations were within the specifications. The specifications and results of the [^68^Ga]Ga-NODAGA-E[c(RGDyK)]_2_ preparations are listed in Supplemental Table S1.

### 3.3. Patient Safety and Dosimetry

The mean and standard deviation of the administered mass of [^68^Ga]Ga-NODAGA-E[c(RGDyK)]_2_ were 23.4 ± 6.4 µg (range, 12.6–35.8 µg). The mean and standard deviation of the administered activity were 184.4 ± 38.4 MBq (range, 97.3–220 MBq). There were no adverse or clinically detectable pharmacologic effects in any of the 10 subjects. No significant changes in vital signs or the results of laboratory studies or electrocardiograms were observed (Supplemental Table S2). No acute or long-term effects on blood parameters or organ function were observed during or after this study (Supplemental Figure S1).

The highest radiation dose was received by the urinary bladder wall (0.126 mSv/MBq) followed by the thyroid and kidneys (0.066 and 0.063 mSv/MBq, respectively) (Table 2). The effective dose was 0.022 mSv/MBq or 4.4 mSv for the intended administered activity dose of 200 MBq (mean value for male and female according to ICRP103 [20]).

### 3.4. Biodistribution and Pharmacokinetics

**Figure 2:**
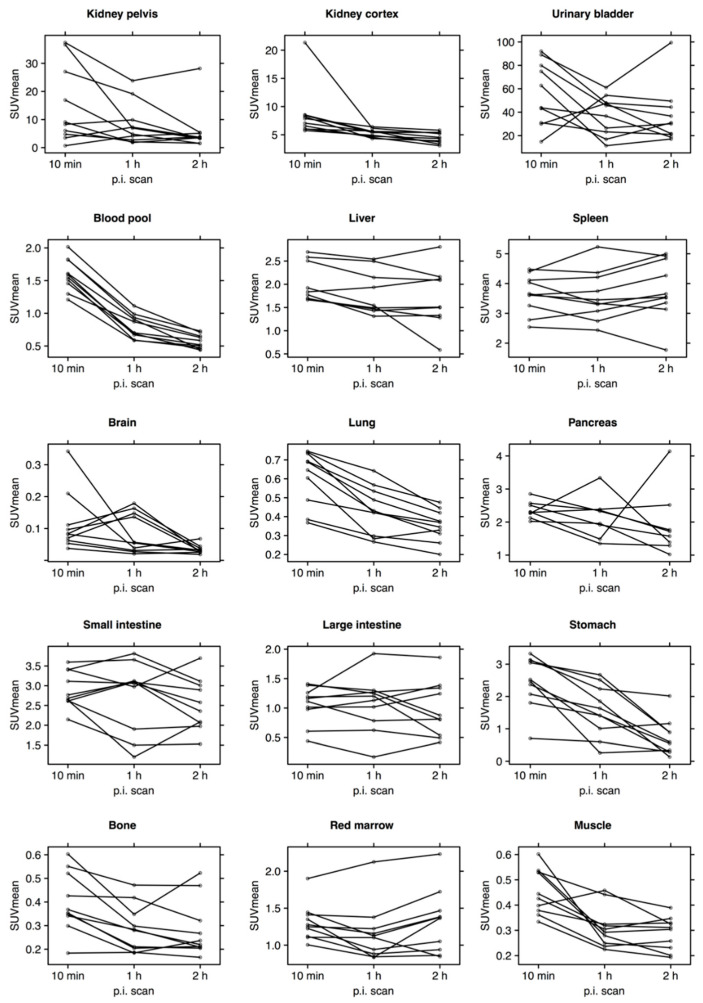
SUV_mean_ in blood and major organs plotted individually for all patients (*n* = 10). For each patient, VOIs were drawn on selected organs/tissues of interest at all three consecutive PET scans. p.i.: post injection.

Decay-corrected SUV_mean_ in blood and major organs is plotted individually for all patients in Figure 2. The kidneys were the primary excretion route, whereas only little excretion was observed through the hepatobiliary/gastrointestinal tract. There was a relatively high, but decreasing, activity in the blood pool. Brain, lungs, bone and muscle showed almost no activity. 

**Figure 3:**
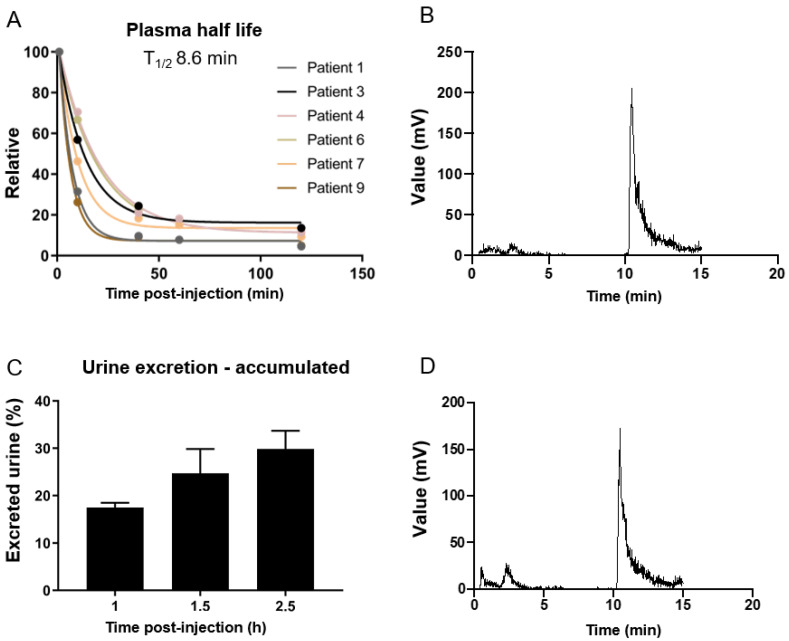
(**A**) Relative time-dependent activity concentrations in plasma. Plasma half-life was estimated to 8.6 min calculated from the half-life from each patient [3.9–14.0 min]. (**B**) A typical example of a plasma sample 10 min after injection analyzed by HPLC showing two unknown plasma metabolites. (**C**) Time-dependent excretion of accumulated activity in urine is displayed. (**D**) A typical example of a urine sample 60 min after injection analyzed by HPLC showing two unknown plasma metabolites.

Blood and urine from seven patients in this study were used for investigation of the plasma pharmacokinetics of [^68^Ga]Ga-NODAGA-E[c(RGDyK)]_2_. Time points for PET scans, blood and urine samples are listed in Supplemental Table S3. A plasma half-life of 8.6 min was found and quantitative analysis of plasma with reversed-phase HPLC showed two unknown polar metabolites (Figure 3).

### 3.5. Tumor Uptake of [^68^Ga]Ga-NODAGA-E[c(RGDyK)]_2_ and Target Validation

**Figure 4:**
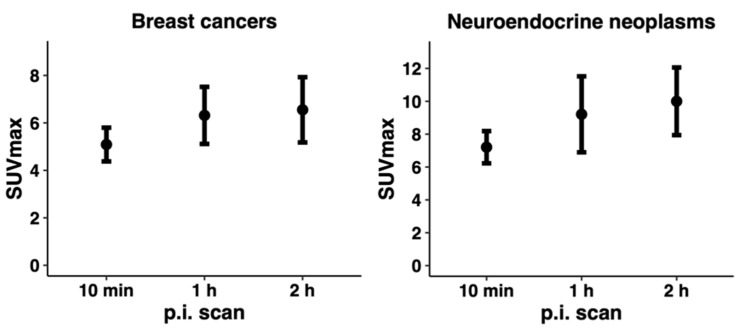
Tumor SUV_max_. Mean value of SUV_max_ ± SEM for all time points (10 min, 1 h and 2 h p.i.) for breast cancer and neuroendocrine neoplasms is illustrated. A rapid accumulation of tracer was observed, and the uptake remained stable over time.

The tracer demonstrated stable tumor retention and a satisfactory image contrast. On qualitative image analysis, tumors were clearly visualized at the first scan 10 min p.i., and the uptake remained relatively stable over time at the PET 1 and 2 h p.i. (Figure 4). All patients with NEN and BC showed tracer uptake in the primary tumor; however, the amount varied in both disease entities (Table 3). Primary tumor-to-organ ratios are shown in Table 4. 

**Figure 5:**
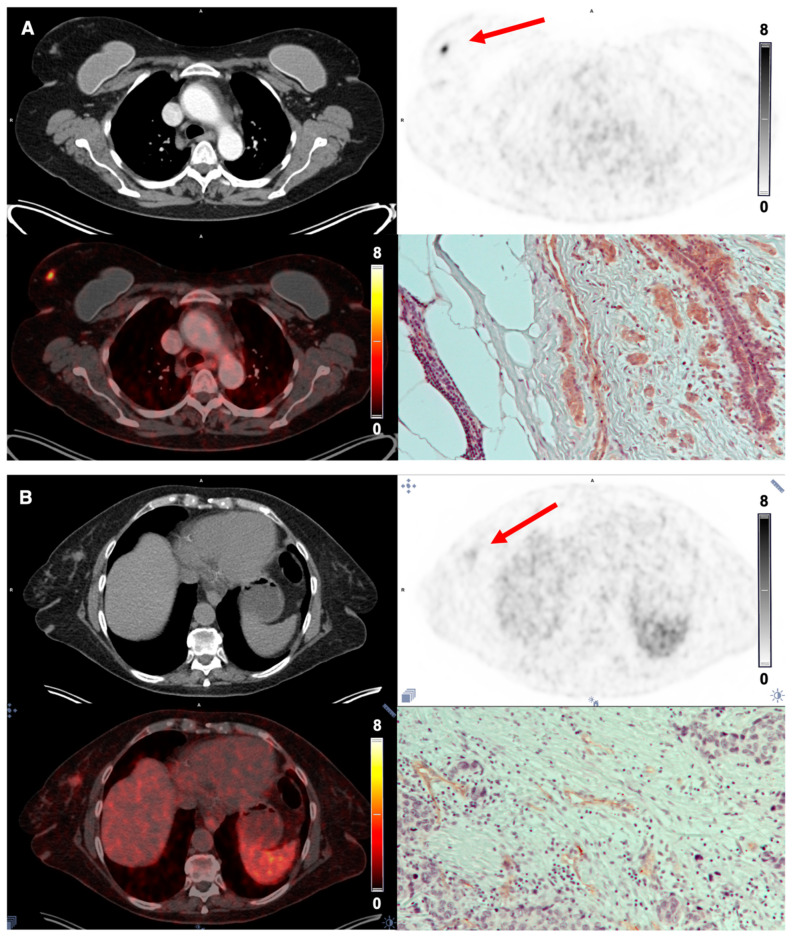
[^68^Ga]Ga-NODAGA-E[c(RGDyK)]_2_ PET imaging in breast cancer. (**A**) Representative transverse CT, PET (1 h p.i.) and fused PET/CT images of primary tumor lesion (red arrow) with a high uptake of [^68^Ga]Ga-NODAGA-E[c(RGDyK)]_2_ (patient 6) and immunohistochemistry staining for integrin α_v_β_3_ in primary tumor showing intense staining. (**B**) CT, PET (1 h p.i.) and PET/CT of primary tumor lesion with a low uptake of tracer (patient 7) and immunohistochemistry staining confirming low intensity of integrin α_v_β_3_ staining.

**Figure 6:**
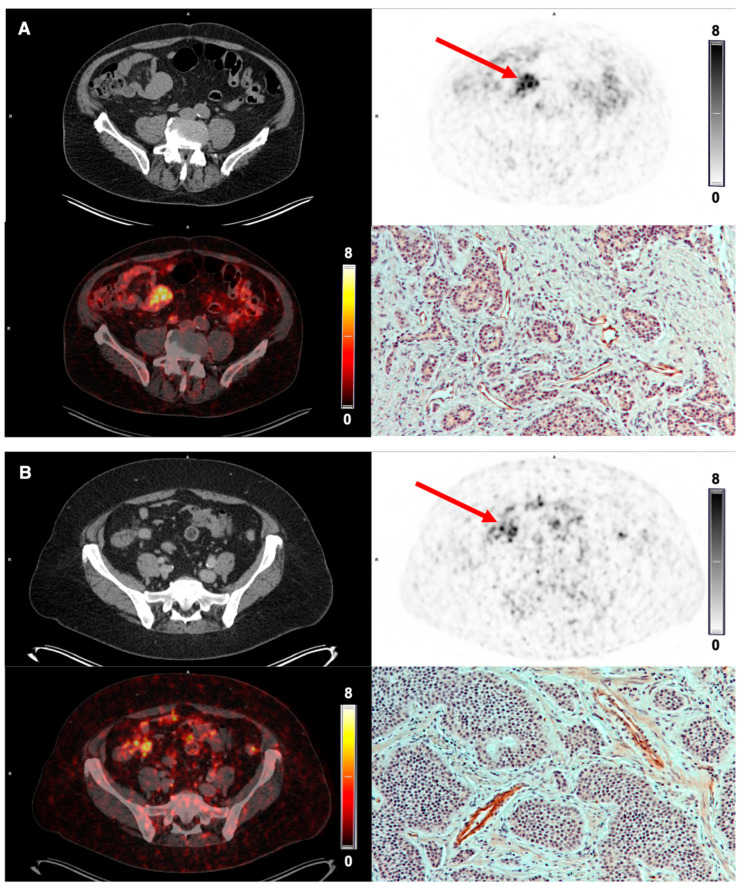
[^68^Ga]Ga-NODAGA-E[c(RGDyK)]_2_ PET imaging in NET. (**A**) Representative transverse CT, PET (1 h p.i.) and fused PET/CT images of primary tumor lesion (red arrow) with a high uptake of [^68^Ga]Ga-NODAGA-E[c(RGDyK)]_2_ in small intestine primary tumor (patient 2) and high intensity immunohistochemistry staining for integrin α_v_β_3_. (**B**) Patient 3 also displays a high uptake of tracer in the terminal ileum primary tumor and a corresponding high intensity of integrin α_v_β_3_ immunohistochemistry staining.

Due to the low background uptake in the normal breast tissue, the primary tumors in patients with BC were visualized clearly, whereas the intestine displayed a slightly higher background uptake for imaging of NEN (Figure 5 and Figure 6). In general, BC displayed a homogeneous tumor uptake of [^68^Ga]Ga-NODAGA-E[c(RGDyK)]_2_, while NEN demonstrated a more heterogeneous uptake. 

A gradual increase in integrin α_v_β_3_-stained blood vessels and tumor cells was seen with increasing tracer uptake, i.e., increasing SUV_max_/SUV_mean_ for both BC and NEN. In Figure 5 and Figure 6, PET images and IHC staining intensity in two patients with BC and two patients with NEN are shown. PET images for the remaining patients are available in Supplemental Figure S2. 

## 4. Discussion

Here, we present the results of our first-in-human phase I study evaluating the safety, biodistribution and dosimetry of [^68^Ga]Ga-NODAGA-E[c(RGDyK)]_2_ PET imaging of integrin α_v_β_3_ in patients with NEN or BC. [^68^Ga]Ga-NODAGA-E[c(RGDyK)]_2_ imaging was safe and no adverse events were observed. Patients did not report any changes in well-being, and no significant changes in vital parameters, electrocardiogram or blood tests (hematology, liver and kidney function) were registered. Biodistribution analysis showed that the kidneys were the primary excretion route, and that hepatobiliary excretion was limited. The effective dose was 0.022 mSv/MBq equaling 4.4 mSv at an injected activity of 200 MBq. Thus, the effective dose is less than the effective dose that is received from a standard ^18^F-FDG PET scan [21].

A secondary objective of our study was to assess [^68^Ga]Ga-NODAGA-E[c(RGDyK)]_2_ uptake in tumors. We observed a higher tracer uptake at all imaging time points in NENs, with a mean SUV_max_ 7.21 (10 min p.i, range 4.53–10.36), 9.60 (1 h p.i, range 4.55–17.70) and 10.00 (2 h p.i., range 5.70–15.35), whereas the mean SUV_max_ in BC was 5.09 (10 min p.i., range 3.05–7.09), 6.32 (1 h p.i., range 2.29–8.75) and 6.55 (2 h p.i., range 2.66–10.53). In both cancer types, we observed a continually increased tumor uptake within the first hour leveling off and stabilizing thereafter, indicating a favorable time point for imaging at 1 h p.i. This was also the case regarding tumor-to-organ ratios, where tumor-to-muscle and tumor-to-liver ratios peaked at 1 h p.i. BC seemed to display a homogeneous tracer uptake, whereas the uptake in NEN was more heterogeneous. However, this may partly be explained by variation in tumor size as the cases with BC and NEN display a median tumor size of 1.4 cm (range 0.9–6.0 cm) and 4.9 (range 4.4–16.0 cm), respectively. Additionally, target validation by comparison of tumor uptake of [^68^Ga]Ga-NODAGA-E[c(RGDyK)]_2_ and tissue expression of integrin α_v_β_3_ was performed. By visual analysis, a correlation between the intensity of integrin α_v_β_3_ staining and tracer uptake was observed. The staining intensity was evaluated visually since no formal scoring system for integrin α_v_β_3_ staining has been developed and due to the low number of patients. Additionally evident from the integrin α_v_β_3_ staining is the fact that α_v_β_3_ is expressed at both newly formed vessels, but also tumor cells. This is in line with previously published immunohistochemical data showing that α_v_β_3_ is indeed expressed on endothelium of neo-vessels as well as on tumor cells [2]. Comparison with other RGD-based PET tracers is complicated by the small sample size of this and other studies, as well as differences in the type of cancer investigated, disease stage and treatments. Previously, other clinically tested PET tracers have reported predominantly renal clearance and moderate tracer uptake in liver, spleen, and intestines [22]. The data presented here for [^68^Ga]Ga-NODAGA-E[c(RGDyK)]_2_ are in line with this, i.e., predominantly renal clearance and moderate uptake in liver, spleen and intestines. When comparing the uptake of PET tracers in tumors, the uptake varies both within the same cancer entities as well as between different cancer entities. In the largest study of BC (*n* = 42), the dimeric RGD-based PET tracer ^18^F-Alfatide II had a mean SUV_max_ of 3.77 ± 1.78 [23]. In the current study, we found a numerically higher tracer uptake in patients with BC (mean SUV_max_ of 6.2 at 1 h p.i.). In our study, patient no. 7 had a triple-negative BC with regional lymph node metastases. Surprisingly, this tumor displayed a low tracer uptake, whereas patient no. 6 with a localized ER and HER2-positive tumor had a high tracer uptake. This was also observed for ^18^F-Alfatide II [23]. To the best of our knowledge, imaging of patients with NEN with RGD tracers has not been reported previously, although different combined tracers with, e.g., RGD and a somatostatin analog have been reported [24]. Multimerization of the RGD motif increases binding avidity, while also increasing radiation of the kidneys due to renal retention [7]. Recently, a study of 10 patients with head and neck squamous cell carcinoma reported mean SUV_max_ of 3.9 ± 1.1 in the primary tumor when applying the monomeric [^68^Ga]Ga-NODAGA-RGDyK [25]. A dosimetry analysis showed an effective dose of 19.8 µSv/MBq and kidney radiation of 0.046 mGy/MBq [26] in comparison with an effective dose of 22 µSv/MBq and kidney radiation of 0.063 mGy/MBq present in the current study. 

Overall, the data of the current phase I trial are encouraging for proceeding with additional testing of [^68^Ga]Ga-NODAGA-E[c(RGDyK)]_2_ for in vivo whole-body assessment of integrin α_v_β_3_ and evaluation of possible clinical implications. While cilengitide targeting α_v_β_3_/α_v_β_5_ revealed a dose-dependent effect with an unintended pro-angiogenic effects at lower concentrations, leading to the overall failure in large trials, newer specific α_v_β_3_ ligands (TDI-3761 and TDI-4161) have shown promising results [6]. Our data underline the varying degree of α_v_β_3_ expression between different cancers, in *casu* BC and NEN. Furthermore, patients that may present with similar disease state based on routine clinical biomarkers, e.g., Ki67% and ER/HER2 receptor status, can show large variation in α_v_β_3_ expression. Accordingly, e.g., patients no. 1 and 2 both had an approximately 4 cm primary NEN in the small intestine, metastatic disease and a Ki67 index of 2%. However, although comparable clinical characteristics, patient no. 2 exhibited the highest SUV_max_ among all of the NEN patients at all time points (SUV_max_: 10.36–17.70), whereas patient no. 1 had the lowest SUV_max_ (SUV_max_: 4.53–5.70). This underscores that in future clinical trials of new selective α_v_β_3_-targeting therapies, patient selection with companion diagnostics α_v_β_3_ integrin imaging may be important to enroll only patients with high levels of integrin expression, thus improving the likelihood of demonstrating a treatment effect. Ultimately, [^68^Ga]Ga-NODAGA-E[c(RGDyK)]_2_ PET may therefore guide decisions on treatments targeting integrin α_v_β_3_ as well as follow-up for monitoring response to treatment [27]. Furthermore, given the relationship between integrin α_v_β_3_ and tumor growth, invasion/metastasis, and angiogenesis, risk stratification of patients may also become possible by means of [^68^Ga]Ga-NODAGA-E[c(RGDyK)]_2_ PET [2]. 

## 5. Conclusions

Based on the present phase I study, we conclude that PET imaging with the tracer [^68^Ga]Ga-NODAGA-E[c(RGDyK)]_2_ is safe and well tolerated. The tracer exhibited a low effective dose and good imaging contrast with variable tumor uptake in NEN and BC probably reflecting inter-individual differences in expression of integrin α_v_β_3_. We suggest that the PET tracer may become a promising tool for early identification of patients eligible for treatments targeting integrin α_v_β_3_ and for risk stratification of patients. 

## 6. Patents

Malene Brandt-Larsen, Jacob Madsen and Andreas Kjaer are inventors/hold IPR on a patent application: “68Ga- and 64Cu -NODAGA-E[c(RGDyK)]2 for use as pet tracers in the imaging of angiogenesis in humans” (WO2019091534A1). No other potential conflicts of interest relevant to this article exist.

## Figures and Tables

**Table 1:** Patient characteristics.

	Patient No.									
Characteristics	1	2	3	4	5	6	7	8	9	10
**Sex**	Male	Male	Female	Female	Male	Female	Female	Female	Female	Male
**Age (y)**	69	79	55	67	58	58	68	68	63	52
**Cancer type**	Neuroendocrine	Neuroendocrine	Neuroendocrine	Breast	Neuroendocrine	Breast	Breast	Breast	Breast	Neuroendocrine
**Stage/grade**	PT in small intestine, metastases in the liver and mesentery	PT in small intestine, metastasis in the mesentery	PT in terminal ileum/coecum	PT left breast	PT not identified, liver metastasis	PT in right breast, SN without metastases	PT in right breast, metastases in 3/14 LN, no distant metastases	PT in left breast, SN without metastases	PT in left breast, SN without metastases	PT in pancreas, liver-, bone, and lymph node metastases
**Biomarker status**	Ki67 2% *	Ki67 2%	Ki67 1%	ER 100%, HER2 borderline	Ki67 14%	ER 100%, HER2 neg.	ER neg., HER2 neg.	ER 100%, HER2 neg.	ER 100%, HER2 neg.	Ki67 25% *
**Concurrent cancer treatment**	Lanreotid	Lanreotid	Lanreotid	None	None	None	None	None	None	None
**Days from PET scan to biopsy/operation**	6	14	30	5	18	6	1	5	5	NA
**Tissue**	NA	Fresh frozen, later paraffin embedded	Fresh frozen, later paraffin embedded	Paraffin embedded	Paraffin embedded	Paraffin embedded	Paraffin embedded	Paraffin embedded	Paraffin embedded	NA

Abbreviations: ER: estrogen receptor. HER2: human epidermal growth factor receptor 2. LN: lymph node. NA: not available. PT: primary tumor. SN: sentinel node. * Obtained from biopsy at time of diagnosis.

**Table 2:** [^68^Ga]Ga-NODAGA-E[c(RGDyK)]_2_ PET dosimetry.

Organ/Tissue	Mean Absorbed Dose (mGy/MBq)
Adrenals	0.02450
Brain	0.00252
Breasts	0.01050
Esophagus	0.01120
Eyes	0.00929
Gallbladder Wall	0.01430
Left Colon	0.01360
Small Intestine	0.06030
Stomach Wall	0.02630
Right Colon	0.01310
Rectum	0.01510
Heart Wall	0.01460
Kidneys	0.06270
Liver	0.02790
Lungs	0.00792
Ovaries	0.01540
Pancreas	0.01440
Prostate	0.01330
Salivary Glands	0.00996
Red Marrow	0.01500
Osteogenic Cells	0.01360
Spleen	0.05040
Testes	0.01920
Thymus	0.01100
Thyroid	0.06630
Urinary Bladder Wall	0.12600
Uterus	0.01800
Total Body	0.01330
**Effective Dose (mSv/MBq)**	**0.02180**

Mean absorbed dose per unit administered activity (mGy/MBq) for major organs was derived from serial whole-body PET scans performed at 10 min, 1 h and 2 h after a single injection of [^68^Ga]Ga-NODAGA-E[c(RGDyK)]_2_ using VOI-based time activity data.

**Table 3:** Summary of [^68^Ga]Ga-NODAGA-E[c(RGDyK)]_2_ PET/CT.

Patient No.	Tumor Type	Tumor Size	Qualitative PET Uptake	SUV_max_			SUV_mean_		
				PET_10_	PET_1h_	PET_2h_	PET_10_	PET_1h_	PET_2h_
1	NEN	4.4 cm	Heterogeneous	4.53	4.55	5.70	2.58	2.37	2.93
2	NEN	4.9 cm	Heterogeneous	10.36	17.70	14.32	5.31	8.74	7.73
3	NEN	4.4 cm	Heterogeneous	7.85	8.77	15.35	4.10	4.48	7.86
4	BC	6 cm	Heterogeneous	6.18	8.75	10.53	3.26	4.52	5.44
5	NEN	16 cm *	Heterogeneous	7.39	9.39	8.83	2.93	3.2	3.15
6	BC	1.1 cm	Homogeneous	4.88	7.15	8.02	4.59	6.75	6.79
7	BC	1.4 cm	Homogeneous	3.05	2.29	2.66	1.67	1.30	1.94
8	BC	1.8 cm	Homogeneous	7.09	8.40	7.10	4.18	4.70	4.04
9	BC	0.9 cm	Homogeneous	4.24	4.99	4.45	2.36	2.54	2.58
10	NEN	10 cm	Heterogeneous	5.90	7.58	5.80	3.27	3.69	3.05

Readouts of primary tumor SUV_max_ and SUV_mean_ for all patients at all time points. Tumor size is based on the largest diameter of primary tumor on CT. * SUV_max_/SUV_mean_ values and tumor size of liver metastasis as location of primary neuroendocrine tumor was unknown. BC: breast cancer. NEN: neuroendocrine neoplasm PET_10_: PET 10 min after injection. PET_1h_: PET 1 h after injection. PET_2h_: PET 2 h after injection.

**Table 4:** Tumor-to-organ ratios for patients with breast cancer or neuroendocrine neoplasms.

	PET 10 min p.i.			PET 1 h p.i.			PET 2 h p.i.		
	BC	NEN	All	BC	NEN	All	BC	NEN	All
Tumor to blood	2.79 (0.45)	3.96 (0.61)	3.37 (0.40)	5.72 (1.27)	9.40 (2.06)	7.56 (1.30)	12.1 (4.11)	11.4 (1.81)	11.7 (2.12)
Tumor to liver	2.37 (0.43)	2.58 (0.30)	2.48 (0.25)	3.18 (0.67)	3.21 (0.69)	3.20 (0.45)	2.67 (0.55)	2.89 (0.36)	2.78 (0.31)
Tumor to kidney	0.60 (0.14)	0.81 (0.16)	0.70 (0.11)	1.07 (0.24)	1.48 (0.26)	1.27 (0.18)	1.08 (0.23)	1.56 (0.24)	1.32 (0.18)
Tumor to muscle	7.11 (1.42)	10.2 (1.81)	8.64 (1.20)	11.9 (4.36)	15.5 (4.08)	13.7 (2.88)	7.40 (2.68)	11.4 (3.35)	9.42 (2.13)

Tumor-to-organ ratios (Tumor lesion SUV_max_/Organ SUV_mean_) are shown as the mean (standard error of mean). All: BC + NEN; BC: breast cancer (*n* = 5); NEN: neuroendocrine neoplasm (*n* = 5); p.i.: post injection.

## Data Availability

Data are not publicly available due to protection of personal data and medical confidentiality.

## Supplementary Materials

The following supporting information can be downloaded at: https://www.mdpi.com/article/10.3390/diagnostics12040851/s1. References [17,18,20] are cited in the supplementary materials. Description of Section S1 quality control of [^68^Ga]Ga-NODAGA-E[c(RGDyK)]_2_ and Section S2 dosimetry; Figure S1: Laboratory tests before and after injection of [^68^Ga]Ga-NODAGA-E[c(RGDyK)]_2_; Figure S2: [^68^Ga]Ga-NODAGA-E[c(RGDyK)]2 PET/CT images; Table S1: Specifications and results of the [^68^Ga]Ga-NODAGA-E[c(RGDyK)]2 preparations; Table S2: Vital parameters during PET scans; Table S3: Time points for PET scans, blood and urine samples.
