# Supplementary material for: First-in-Human Study of [68Ga]Ga-NODAGA-E[c(RGDyK)]2 PET for Integrin αvβ3 Imaging in Patients with Breast Cancer and Neuroendocrine Neoplasms: Safety, Dosimetry and Tumor Imaging Ability
Source: Diagnostics (Basel). 2022 Mar 30;12(4):851. doi: 10.3390/diagnostics12040851 (PMC9027224; doi:10.3390/diagnostics12040851)

diagnostics

MDPI

Supplemental material

First-In-Human Study of [^{68}Ga]Ga-NODAGA-E[c(RGDyK)]$_2$ PET for Integrin $\alpha_v\beta_3$ Imaging in Patients with Breast Cancer and Neuroendocrine Neoplasms: Safety, Dosimetry and Tumor Imaging Ability

Malene Martini Clausen [1,2,3,†], Esben Andreas Carlsen [1,3,†], Camilla Christensen [1,3], Jacob Madsen [1,3], Malene Brandt-Larsen [1,3], Thomas Levin Klausen [1,3], Søren Holm [1,3], Annika Loft [1,3], Anne Kiil Berthelsen [1,3], Niels Kroman [4], Ulrich Knigge [3,5] and Andreas Kjaer [1,3,*]

1 Department of Clinical Physiology and Nuclear Medicine & Cluster for Molecular Imaging, Copenhagen University Hospital—Rigshospitalet & Department of Biomedical Sciences, University of Copenhagen, 2100 Copenhagen, Denmark; malene.martini.clausen@regionh.dk (M.M.C.); esben.andreas.carlsen.01@regionh.dk (E.A.C.); cch@minervaimaging.com (C.C.); jacob.madsen@regionh.dk (J.M.); bbqdk@leo-pharma.com (M.B.-L.); thomas.levin.klausen@regionh.dk (T.L.K.); soeren.holm@regionh.dk (S.H.); annika.loft.jakobsen@regionh.dk (A.L.); anne.kiil.berthelsen@regionh.dk (A.K.B.)

2 Department of Oncology, Copenhagen University Hospital—Rigshospitalet, 2100 Copenhagen, Denmark

3 ENETS Neuroendocrine Tumor Center of Excellence, Copenhagen University Hospital—Rigshospitalet, 2100 Copenhagen, Denmark

4 Department of Breast Surgery, Copenhagen University Hospital—Herlev/Gentofte Hospital, 2730 Herlev, Denmark; niels.kroman@regionh.dk

5 Departments of Clinical Endocrinology and Surgical Gastroenterology, Copenhagen University Hospital—Rigshospitalet, 2100 Copenhagen, Denmark; ulrich.peter.knigge@regionh.dk

* Correspondence: akjaer@sund.ku.dk

† Shared first authors. These authors contributed equally to this work

Supplementary Material

Section S1. Quality control of [^{68}Ga]Ga-NODAGA-E[c(RGDyK)]$_2$

For analysis, a high-performance liquid chromatograph (HPLC) (Ultimate 3000; Dionex) was used with a 2.6 µm, 100Å, 50 × 4.6 mm C18 column (Kinetex). The mobile phases were: eluent A: 0.1% trifluoroacetic acid in H_2O; eluent B: 0.1% trifluoroacetic acid in MeCN. TLC analysis was performed using iTLC-plates eluted with 77 g/L solution of ammonium acetate in water: methanol (50:50 V/V) The TLC plates were subsequently scanned using a Scan-RAM radio-TLC scanner (LabLogic). Gas chromatography (Shimadzu) analysis was performed on a Zebron ZB-WAX 30 m x 0.53 mm x 1.00 µm column (column temperature: 70ºC, carrier gas: He, flow rate: 4.7 ml/min).

The radiochemical purity was more than 97 % on HPLC, and the amount of unlabeled ^{68}Ga in the product was less than 2 %, as demonstrated by radio–thin layer chromatography (TLC). pH was 4.7 ± 0.1, EtOH content was 4.9 ± 0.6 %, and the product was sterile and free from endotoxins.

See specifications and results in Supplemental Table S1.

Section S2. Dosimetry

Dosimetry was based on the decay-uncorrected image sets from the 3 time-points (10 patients) supplemented with sampled urine-data (7 patients). The following organs were considered: adrenal gland, bone, brain, heart, kidney, liver, lung, red marrow, small in-

testine, spleen, stomach contents, testes, thyroid, and upper large intestine. For each patient, organ, and time-point, tissue activity concentration (kBq/mL) was calculated as the average of the mean values from 3 VOIs. Total activity (per patient, organ and time) was estimated by multiplying these average values by organ masses of the OLINDA male adult phantom [1]. Activity values were normalized to 1MBq by dividing with injected activity and scaled with the ratio of actual patient weight to the weight of the standard model (73 kg). As a surrogate for red marrow activity, VOIs were drawn encompassing large portions of the L3–L5 vertebrae. Time integrated activity coefficients (TIAC, formerly known as *residence time*; unit *h*) for each patient and organ was determined by numerical integration up to the third (last) data point and analytical extrapolation to infinity assuming only physical decay. Piecewise linearity was assumed from time zero up to the second data point and a mono-exponential was used between the second and third data points. The resulting organ TIACs were averaged over patients. All data were entered into OLINDA/EXM 2.0 software (Vanderbilt University, and HERMES Medical Solutions) [2].

Urine was collected immediately after each scan in pre-weighted plastic bottles and for each individual voiding the weight (volume) was measured and a 500 μL sample was drawn for activity measurement in a calibrated gamma well counter (Cobra II TM, Gamma Counting Systems; PACKARD, Meriden, CT, USA).The cumulated decay corrected activity (in MBq) of the excreted urine, normalized to 1 MBq of injection, was plotted over time (in Excel) for all 7 subjects and data fitted to a one phase exponential association (exponential growing towards a limit). The resulting limit and half-life were used as input to the bladder voiding model of OLINDA, yielding the TIAC for bladder contents. In concordance with the actual setup a bladder voiding interval of 1 hour was selected.

The value for "remainder tissue" was calculated as the total area (in h) for 1 MBq minus the sum of the organ-specific values (except bladder) minus the value passed to urine (based on the fitted model parameters). The fraction of the latter that is attributed to the bladder depends on the voiding interval.

The output from OLINDA consists of absorbed doses for organs and effective dose with weight factors according to ICRP 103 [3].

Supplement reference list

1. Stabin, M.G.; Siegel, J.A. Physical models and dose factors for use in internal dose assessment. *Health Phys* **2003**, *85*, 294-310, doi:10.1097/00004032-200309000-00006.
2. Stabin, M.G.; Siegel, J.A. RADAR Dose Estimate Report: A Compendium of Radiopharmaceutical Dose Estimates Based on OLINDA/EXM Version 2.0. *J Nucl Med* **2018**, *59*, 154-160, doi:10.2967/jnumed.117.196261.
3. The 2007 Recommendations of the International Commission on Radiological Protection. ICRP publication 103. *Ann ICRP* **2007**, *37*, 1-332, doi:10.1016/j.icrp.2007.10.003.

Supplemental Table S1. Specifications and results of the [^{68}Ga]Ga -NODAGA-E[c(RGDyK)]$_2$ preparations

**Analysis**		**Specification**	**Results (n=10)**
Appearance		Clear and colorless solution, free from visible particles or cloudiness	Clear and colorless solution, free from visible particles or cloudiness
pH		4.0 – 8.0	4.7 (4.5-4.9)
Radioactivity		100 – 800 MBq*	533.3 MBq (197.4-858.9 MBq)
Radiochemical purity	HPLC	> 91%	99.1% (97.7-99.8%)
	iTLC: free [^{68}Ga] + [^{68}Ga]kolloid	≤ 2%	0.5% (0.0-1.6%)
	iTLC: Total radiochemical purity	> 91%	98.5% (96.1-99.5%)
Chemical purity	Other impurities	< 32 µg/patient	0.7 (0.0-4.0)
	Total NODAGA-E[c(RGDyK)]$_2$	< 7.7 µg/mL (maximum injected dose 70 µg/patient)	32.2 µg/patient (18.0-58.0 µg/patient)
	Ethanol	2 - 6 % (w/v)	4.9 (3.3-5.5)
LAL-test**		< 2.5 EU/mL	0.55 EU/mL

*+/- 10% **Performed retrospectively on every batch (not release test)

Supplemental Table S2. Vital parameters during PET scans

**Blood pressure, pulse and oxygen saturation during PET/CT scans**																				
**Time**	**Before**				**1 min post injection**				**10 min post injection**				**1 hour post injection**				**2 hours post injection**			
**Pt**	**SBP**	**DBP**	**P**	**SpO$_2$**	**SBP**	**DPB**	**P**	**SpO$_2$**	**SBP**	**DBP**	**P**	**SpO$_2$**	**SBP**	**DBP**	**P**	**SpO$_2$**	**SBP**	**DBP**	**P**	**SpO$_2$**
1	122	77	57	99	117	60	57	99	109	63	57	96	118	67	51	98	128	70	57	97
2	115	90	73	95	105	64	69	98	93	64	67	95	127	80	77	95	116	76	57	100
3	156	100	72	95	165	92	60	96	149	88	70	96	125	75	65	97	142	83	72	96
4	153	87	80	96	174	90	79	97	156	100	76	95	157	103	89	99	157	96	83	98
5	147	88	87	100	149	84	87	99	133	84	81	97	140	85	87	98	149	88	71	98
6	150	85	77	94	155	83	67	97	143	76	73	96	145	79	77	96	144	88	77	99
7	164	74	66	96	163	69	68	96	147	68	61	97	159	62	68	97	144	68	64	97
8	130	72	67	95	135	78	67	96	134	68	67	96	134	68	68	95	133	70	57	94
9	124	77	57	94	126	83	71	96	118	76	67	97	139	84	57	97	139	82	67	97
10	126	72	61	96	124	72	58	96	125	71	67	97	134	69	63	97	133	71	67	97

Pt: patient number, SBP: systolic blood pressure, DBP: diastolic blood pressure, P: pulse, SpO$_2$: oxygen saturation

Supplemental Table S3. Time points for PET scans, blood and urine samples for individual patients

A. PET scans

Pt	PET 10 min p.i.	PET 1 h p.i.	PET 2 h p.i.
1	11 min. p.i.	1 h 9 min. p.i.	2 h 7 min. p.i.
2	11 min. p.i.	1 h 5 min. p.i.	2 h p.i.
3	14 min. p.i.	1 h 9 min. p.i.	2 h 2 min. p.i.
4	18 min. p.i.	1 h 12 min. p.i.	2 h 10 min. p.i.
5	11 min. p.i.	60 min. p.i.	2 h 1 min. p.i.
6	13 min. p.i.	1 h 4 min. p.i.	2 h 3 min. p.i.
7	14 min. p.i.	1 h 9 min. p.i.	2 h 2 min. p.i.
8	11 min. p.i.	1 h 1 min. p.i.	2 h 1 min. p.i.
9	11 min. p.i.	60 min. p.i.	2 h p.i.
10	14 min. p.i.	1 h 3 min. p.i.	1 h 59 min. p.i.

B. Pharmacokinetics - Blood

Pt	1 min. p.i.	10 min. p.i.	30-40 min. p.i.	1 h p.i.
1	2 min. p.i.	9 min. p.i.	44 min. p.i.	1 h 3 min. p.i.
2	NA	8 min. p.i.	48 min. p.i.	58 min. p.i.
3	1 min. p.i.	10 min. p.i.	48 min. p.i.	NA
4	4 min. p.i.	11 min. p.i.	46 min. p.i.	1 h 2 min. p.i.
5	ND	ND	ND	ND
6	1 min. p.i.	8 min. p.i.	37 min. p.i.	ND
7	2 min. p.i.	8 min. p.i.	38 min. p.i.	1 h 2 min. p.i.
8	ND	ND	ND	ND
9	1 min. p.i.	8 min. p.i.	44 min. p.i.	53 min. p.i.
10	ND	ND	ND	ND

C. Pharmacokinetics - Urine

Pt	30-40 min. p.i.	After PET 1 h p.i.	After PET 2 h p.i.
1	49 min. p.i.	1 h 42 min. p.i.	2 h 46 min. p.i.
2	56 min. p.i.	1 h 41 min. p.i.	2 h 49 min. p.i.
3	50 min. p.i.	1 h 47 min. p.i.	2 h 42 min. p.i.
4	54 min. p.i.	1 h 46 min. p.i.	2 h 48 min. p.i.
5	ND	ND	ND
6	49 min. p.i.	1 h 37 min. p.i.	ND
7	38 min. p.i.	1 h 41 min. p.i.	2 h 33 min. p.i.
8	ND	ND	ND
9	46 min. p.i.	1 h 42 min. p.i.	2 h 54 min. p.i.
10	ND	ND	ND

Pt: patient number, NA: not available, ND: not done, p.i.: post injection.

Supplemental Figure S1. Laboratory tests before and after injection of [68Ga]Ga -NODAGA-E[c(RGDyK)]₂

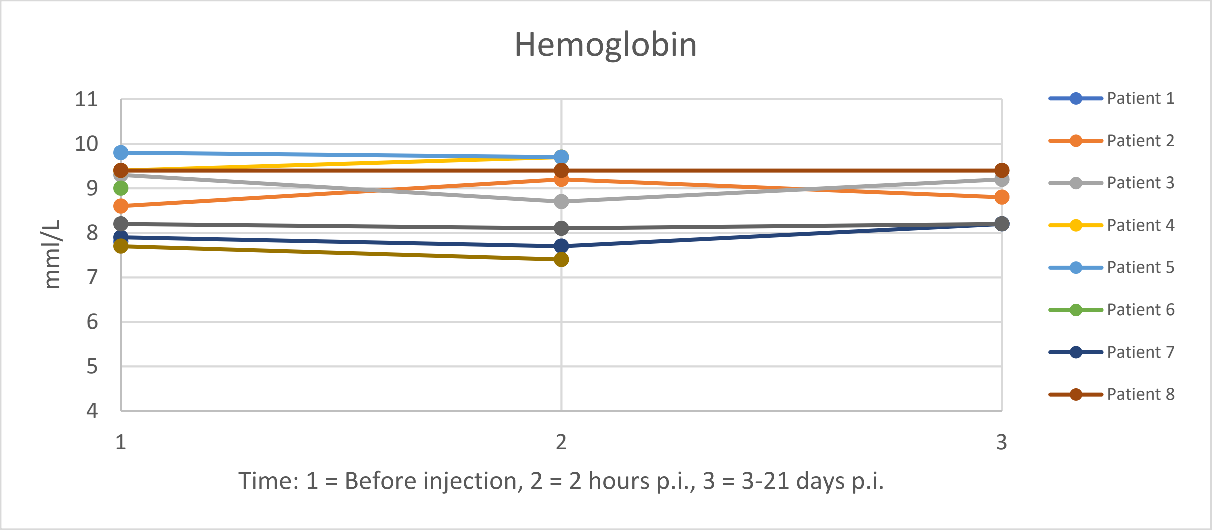

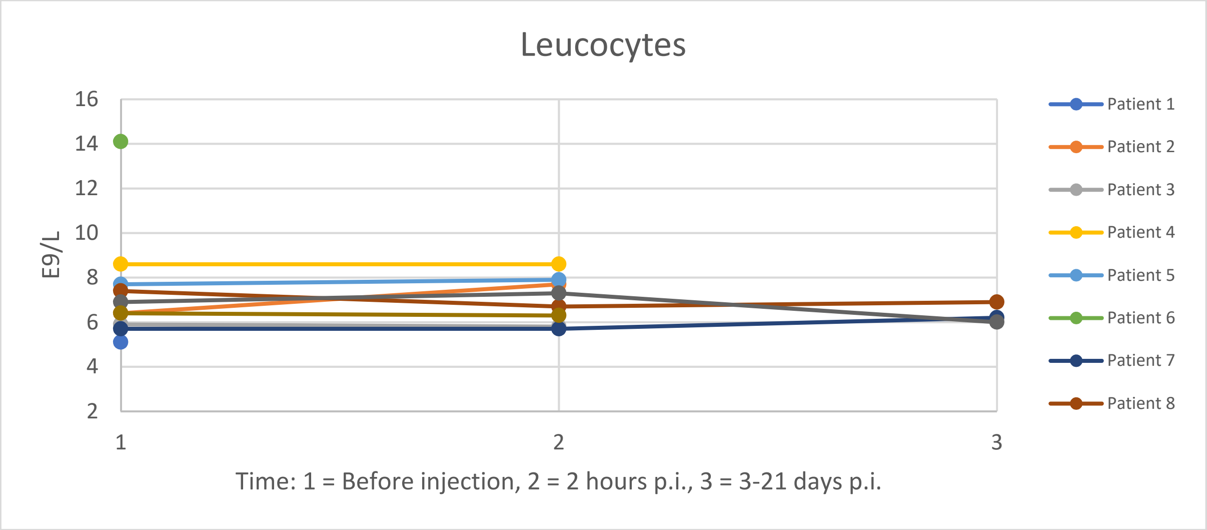

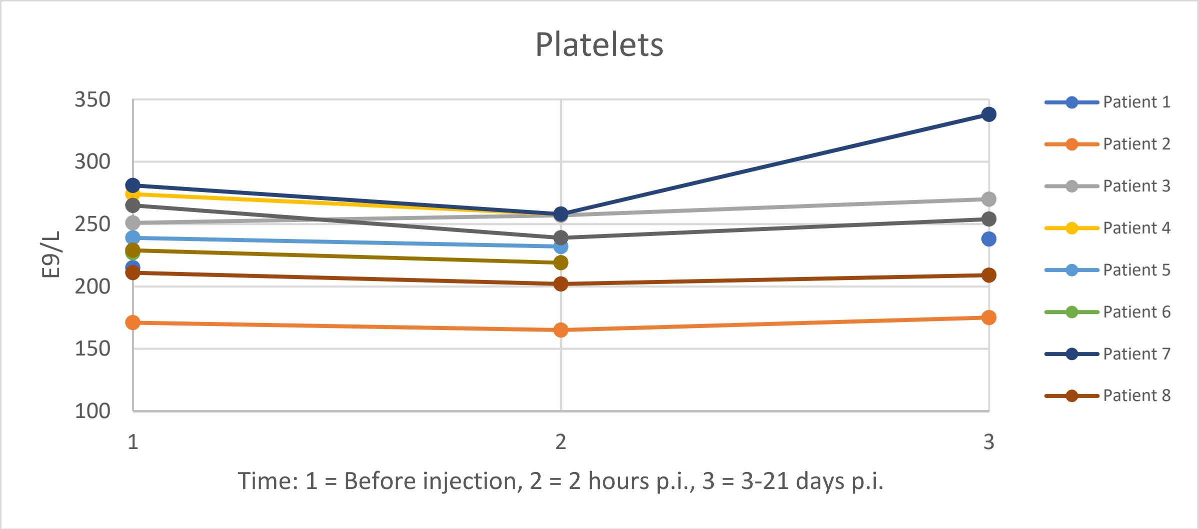

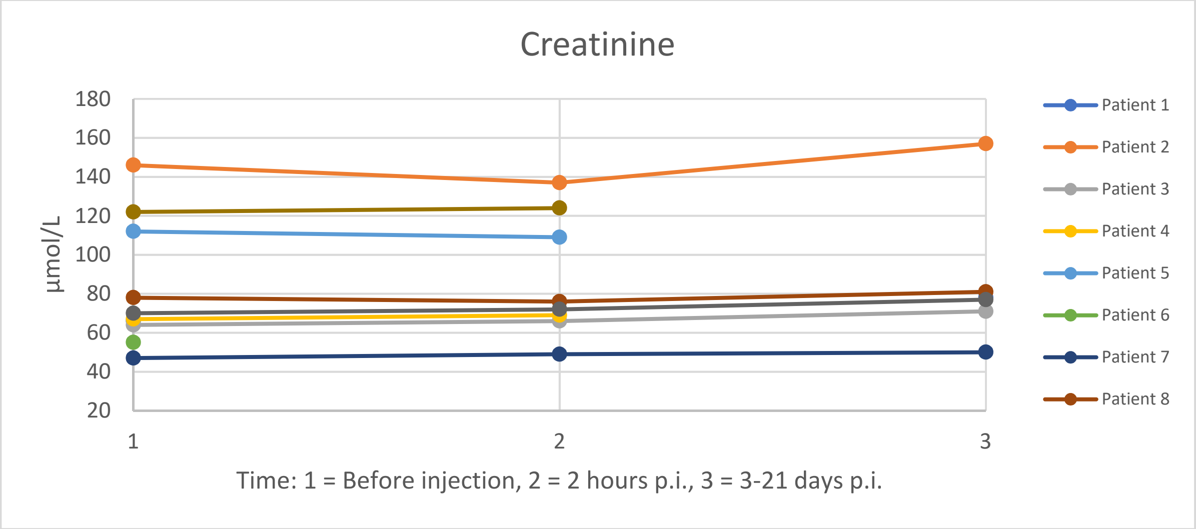
Creatinine
µmol/L
180
160
140
120
100
80
60
40
20
1
2
3
Time: 1 = Before injection, 2 = 2 hours p.i., 3 = 3-21 days p.i.
Patient 1
Patient 2
Patient 3
Patient 4
Patient 5
Patient 6
Patient 7
Patient 8

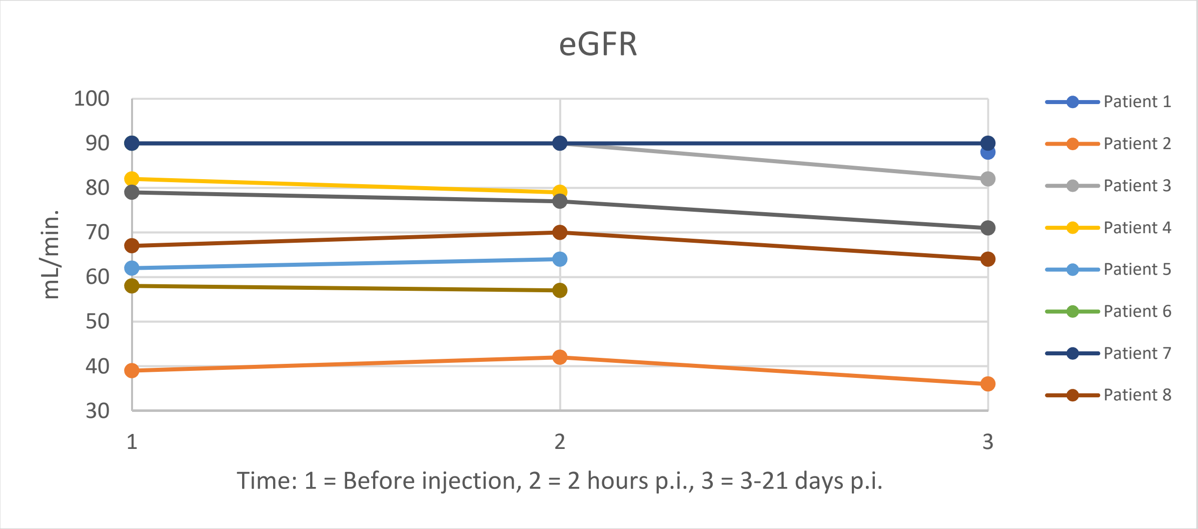
eGFR
mL/min.
100
90
80
70
60
50
40
30
1
2
3
Time: 1 = Before injection, 2 = 2 hours p.i., 3 = 3-21 days p.i.
Patient 1
Patient 2
Patient 3
Patient 4
Patient 5
Patient 6
Patient 7
Patient 8

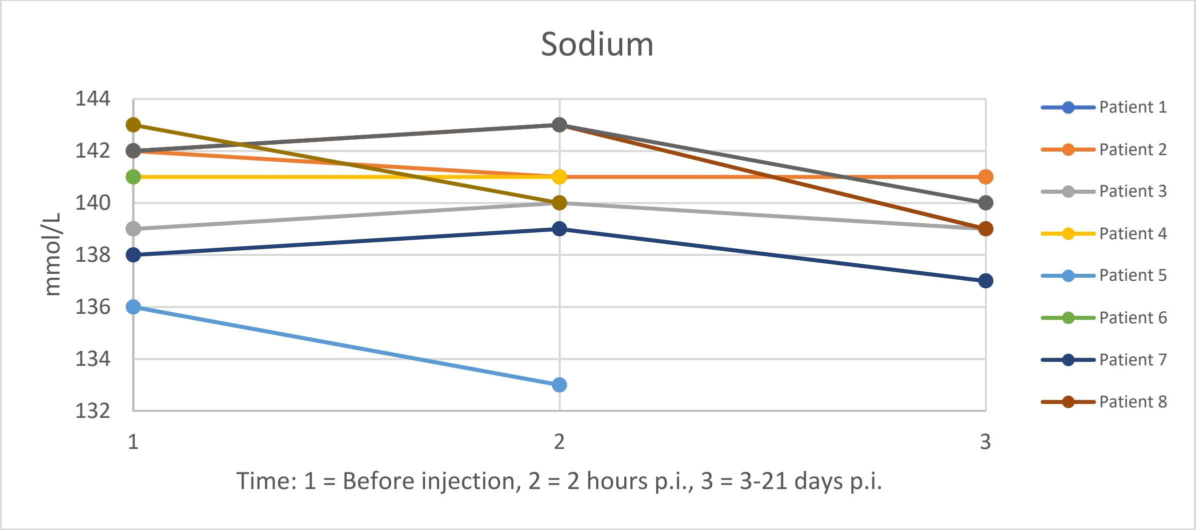
Sodium
mmol/L
144
142
140
138
136
134
132
1
2
3
Time: 1 = Before injection, 2 = 2 hours p.i., 3 = 3-21 days p.i.
Patient 1
Patient 2
Patient 3
Patient 4
Patient 5
Patient 6
Patient 7
Patient 8

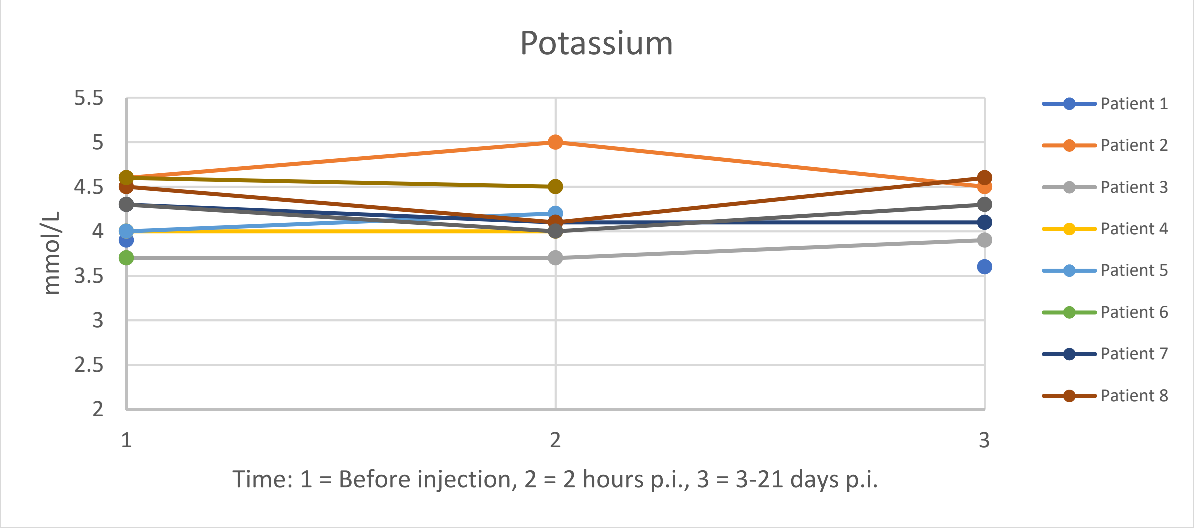
Potassium
mmol/L
5.5
5
4.5
4
3.5
3
2.5
2
1
2
3
Time: 1 = Before injection, 2 = 2 hours p.i., 3 = 3-21 days p.i.
Patient 1
Patient 2
Patient 3
Patient 4
Patient 5
Patient 6
Patient 7
Patient 8

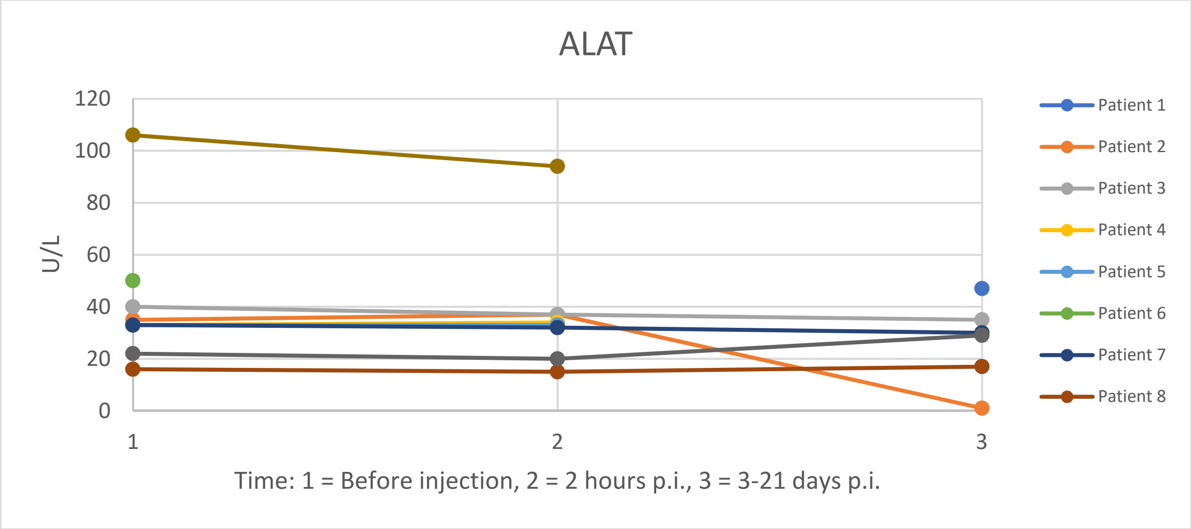
ALAT
U/L
120
100
80
60
40
20
0
1
2
3
Time: 1 = Before injection, 2 = 2 hours p.i., 3 = 3-21 days p.i.
Patient 1
Patient 2
Patient 3
Patient 4
Patient 5
Patient 6
Patient 7
Patient 8

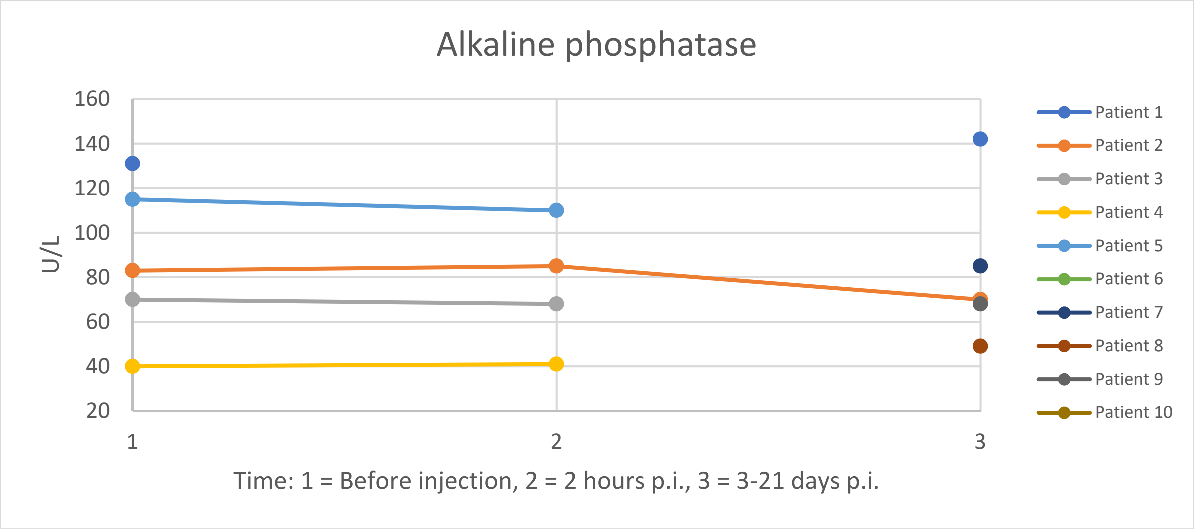
Alkaline phosphatase
U/L
160
140
120
100
80
60
40
20
1
2
3
Time: 1 = Before injection, 2 = 2 hours p.i., 3 = 3-21 days p.i.
Patient 1
Patient 2
Patient 3
Patient 4
Patient 5
Patient 6
Patient 7
Patient 8
Patient 9
Patient 10

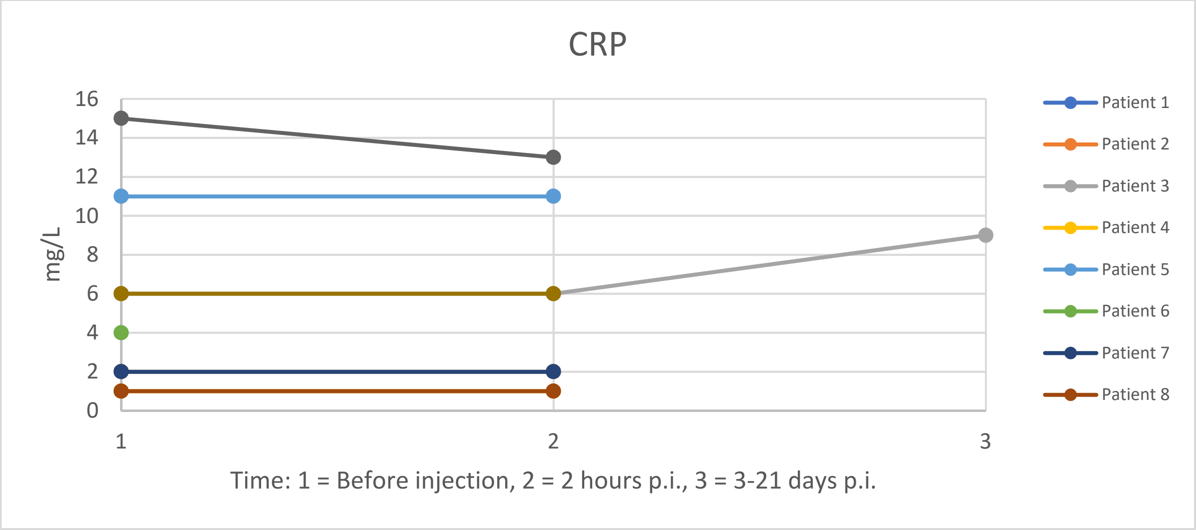
CRP
16
14
12
10
8
6
4
2
0
mg/L
1
2
3
Time: 1 = Before injection, 2 = 2 hours p.i., 3 = 3-21 days p.i.
Patient 1
Patient 2
Patient 3
Patient 4
Patient 5
Patient 6
Patient 7
Patient 8

Supplemental Figure S2. [^{68}Ga]Ga -NODAGA-E[c(RGDyK)]$_2$ PET/CT images. **(A-C):** Neuroendocrine neoplasm (patient no. 1, 5 and 10) **(D-F):** Breast cancer (patient no. 4, 8 and 9)

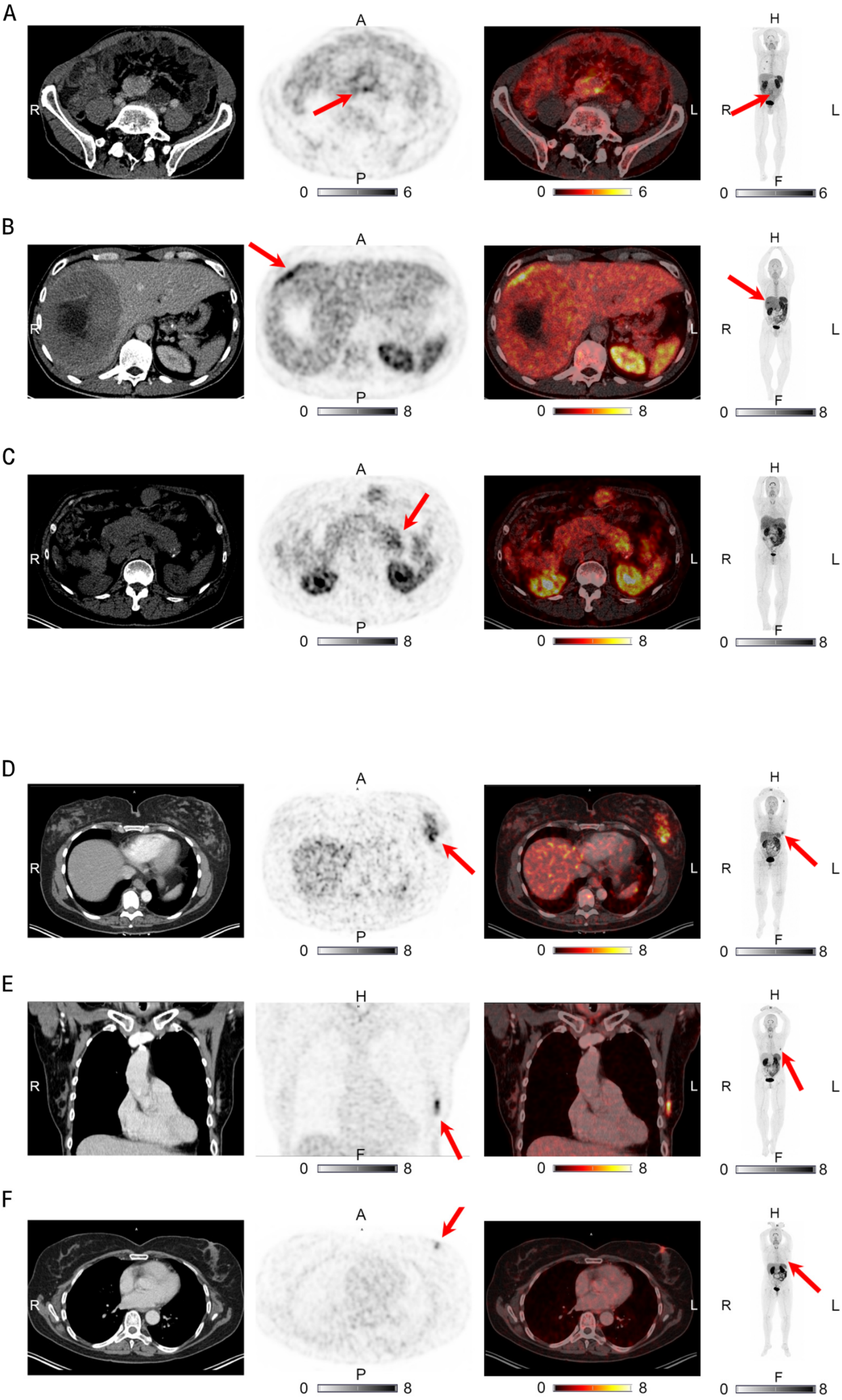

Supplement: Supplementary file 1 [file diagnostics-12-00851-s001.zip › diagnostics-1658458-supplementary.pdf]
